# An Unambiguous Synchronization Scheme for GNSS BOC Signals Based on Reconstructed Correlation Function

**DOI:** 10.3390/s21061982

**Published:** 2021-03-11

**Authors:** Xiyan Sun, Shaojie Song, Yuanfa Ji, Xingli Gan, Suqing Yan, Xizi Jia

**Affiliations:** 1 Guangxi Key Laboratory of Precision Navigation Technology and Application, Guilin University of Electronic Technology, Guilin 541004, China; sunxiyan1@163.com (X.S.); ssj1816977054@163.com (S.S.); yansuqing163@163.com (S.Y.); 18907830034@163.com (X.J.); 2 Information and Communication School, Guilin University of Electronic Technology, Guilin 541004, China; 3 National & Local Joint Engineering Research Center of Satellite Navigation Positioning and Location Service, Guilin 541004, China; 4 School of Information and Electronic Engineering, Zhejiang University of Science and Technology, Hangzhou 310023, China; ganxingli@163.com

**Keywords:** Global Satellite Navigation System (GNSS), binary offset carrier (BOC), synchronization, reconstruction correlation function, unambiguous

## Abstract

Binary offset carrier (BOC) modulation is a new modulation method that has been gradually applied to the Global Satellite Navigation System (GNSS) in recent years. However, due to the multi-peaks in its auto-correlation function (ACF), it will incur a false lock and generate synchronization ambiguous potentially. In this paper, an unambiguous synchronization method based on a reconstructed correlation function is proposed to solve the ambiguity problem. First, through the shape code vector constructed in this paper, the general cross-correlation function (CCF) expression of the BOC modulated signal will be obtained. Based on the features of the signal correlation function, it is decomposed into a matrix form of trigonometric functions. Then, it generates two local signal waves using a specific method, then the proposed method is implemented to obtain a no-side-peak correlation function by reconstructing the cross-correlation between the received signal and the two local signals. Simulations showed that it fully eliminates the side-peak threat and significantly removes the ambiguity during the synchronization of the BOC signals. This paper also gives the improved structure of acquisition and tracking. The detailed theoretical deduction of detection probability and code tracking error is demonstrated, and the corresponding phase discrimination function is given. In terms of de-blurring ability and detection probability performance, the proposed method outperformed other conventional approaches. The tracking performance was superior to the comparison methods and the phase discrimination curve only had a zero-crossing, which successfully removed the false lock points. In addition, in multipath mitigation, it outperformed the ACF of the BOC signal, and performs as well as the autocorrelation side-peak cancellation technique (ASPeCT) for BOC(*kn*,*n*) signals.

## 1. Introduction

In the future, binary offset carrier (BOC) modulation [1,2] will become the focus of design and research in the Global Navigation Satellite System (GNSS). BOC has been applied in some new signals, for example, GPS L1C and Galileo open service (OS) signals. Compared with the conventional binary phase shift keying (BPSK) [3,4], the BOC modulation moves signal energy away from the band center and thus realizes a high level of frequency spectrum separation. In addition, the autocorrelation peak of the BOC modulation signal is sharper than the BPSK, so it is superior to BPSK in the tracking performance and multipath mitigation capability. However, the BOC modulation has some shortcomings, the most severe being the ambiguity problem in synchronization [5]. The auto-correlation function (ACF) of BOC signals has a saw-toothed profile, piecewise linear ACF, which has a multi-peak. Thus, this characteristic will induce multiple zero-crossing points in the discriminator and false locks that may lead to huge and intolerable acquisition and tracking error bias in measurements for the modern GNSS [6,7,8]. The severe ambiguity of synchronization processing of the BOC signals will cause evident impact on the positioning accuracy, which is intolerable to high precision navigation systems [9]. Therefore, how to effectively eliminate the ambiguity in the synchronization process of the BOC modulation signal is a hot issue in the current navigation signal processing field.

Currently, the solutions to tackle the ambiguity problem have been highly focused on by many researchers, and some interesting approaches can be summed up in the following three categories: single sideband algorithm [10,11], ambiguity avoidance detection method [12,13] and side-peaks cancellation (SC) [14,15,16,17,18,19,20,21]. (1) The BPSK-like [10] and modified sideband (MSB) [11] technique are two representative single-sideband algorithms, both of which use band-pass filters to deal BOC signals to obtain two side lobes. However, they will achieve a wider main peak and wholly eliminate all the merits of BOC signal in synchronization. (2) Both the bump-jumping (BJ) [12] and double estimation technique (DET) [13] belong to the ambiguity avoidance detection method. This type of method is used to increase the number of correlators to detect and reduce the tracking error, but it requires a longer detection and recovery time and is not a fit method for weak signal strength to use. (3) The side-peaks cancellation technique refers to an unambiguous correlation function combination method by introducing several local reference signals. The first side-peaks cancellation technique is called SCPC (sub carrier phase cancelation) [14], which performs correlation processing on the received signals with the locally generated in-phase and quadrature signals, and then superimposes the two correlated signals. For SCPC [14], the number of correlators are more than the BPSK-like, but both have the same effect in synchronization performance. Autocorrelation side-peak cancellation technique (ASPeCT) is demonstrated in [15]. This technique completely removes the influence caused by side peaks and keeps the advantage of narrow main peak. However, this method reduces the pull-in range of the code loop. Once the code phase error exceeds its correlation peak width (that is, the width of the main correlation peak of the BOC signal), the tracking loop may lose lock and the Sine-BOC(*n*,*n*) signals are only applicable to this method. The pseudo-correlation function (PCF) technology mentioned in [16] eliminates the edge peaks of the correlation function by constructing two sets of spreading code sequence waveforms that are mirror images of each other, and then combining them non-linearly. The method in [17,18] is only dedicated to Sine-BOC signals. General removing ambiguity via side peak suppression (GRASS) technology [19] is a generalized ambiguity-free capture algorithm proposed for sine-BOC signals, but at the expense of the narrow correlation characteristics of BOC signals. On this basis, [20,21] improved the method, respectively, but the common shortcoming of these two methods is that the edge peak elimination is not thorough enough, and that used in [21] is only suitable for cosine-BOC signals of an even modulation order.

This paper introduces a novel reconstructed correlation function technique of BOC signal according to the shape code vector. The synchronization ambiguity is solved by the designed method in the baseband signal processing of the navigation receiver. The main contributions and research content of this paper are as follows. First, the existing acquisition algorithm that cannot completely eliminate the secondary peak of the correlation function will cause a stable false lock point in the phase discrimination output curve. In this paper, the acquisition ambiguity caused by the above reason will be completely eliminated. Second, aiming at the problem that most acquisition/tracking algorithms can only use one of the sine or cosine phased BOC signals, the proposed synchronization algorithm can effectively act on both signals, which broadens the scope of application of the algorithm. Finally, the proposed technique is proven to have noteworthy multipath mitigation performance compared with several other anti-multipath methods.

The rest of the paper is organized as follows. Section 2 introduces the main characteristics of BOC signals and the ambiguous problem. Section 3 describes the principle of the unambiguous synchronization technique, defines the locally auxiliary signal, demonstrates the unambiguous acquisition structure, and proposes the improved code tracking loop and the phase discrimination function. Section 4 shows the simulation results and performance analysis. This paper will fully demonstrate the superiority of the synchronization algorithm from the following six aspects of de-blurring, detection probability, peak-to average ratio, code tracking accuracy, anti-multipath, and phase discrimination. Section 5 finally reaches a conclusion.

## 2. Binary Offset Carrier (BOC) Modulated Signals

### 2.1. Definitions and Main Characteristics

**Figure 1:**
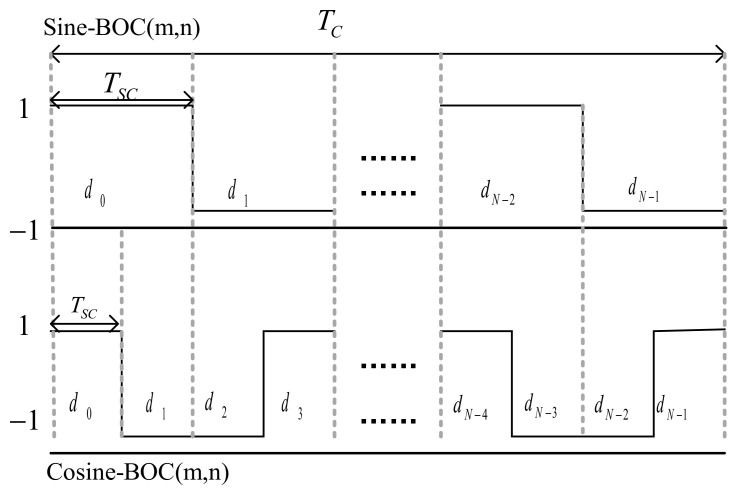
Binary offset carrier (BOC) signals and shape vectors.

BOC modulated signals can be represented as BOC(*m*,*n*), where m is the ratio of the square wave subcarrier frequency to 1.023 MHz and n represents the ratio of spreading code frequency to 1.023 MHz [22]. By definition, the baseband spread spectrum signal can be represented as:

(1) s(t)=c(t)sc(t),

where c(t) is the pseudo random noise code (PRN) and sc(t) is the square wave subcarrier. The two mathematical expressions are as follows:

(2) $$c(t)=\sum_{i=-\infty }^{\infty }C_{i}P_{Tc}(t-iTc)$$

where $C_{i}$ is the symbol of the *i*th chip, the value of $C_{i}$ between −1 and 1; Tc is the chip width of a PRN code; and $P_{Tc}(t)$ is a square wave of the width being Tc and the amplitude being 1.

(3) $$sc(t)=\sum_{j=0}^{N-1}d_{j}P_{Tsc}(t-jTsc)$$

where $P_{Tsc}$ is the rectangular pulse with the period being Tsc, which is the width of one subcarrier pulse, and the amplitude being 1; N represents that one modulated symbol is divided into N segments, each with equal length Tsc. $d_{j}\in \{1,-1\}\;(j=0,\;1,\;2,\cdots ,N-1)$, defines this kind of symbol as a shape symbol. Each shape vector $d=[d_{0}\;d_{1}\;\cdots \;d_{N-1}]$ consists of shape symbols. The BOC signals of sine and cosine can be described by the different shape vectors, as seen in Figure 1.

For Sine-BOC(*m*,*n*), N=2m/n and the shape vector is $d={\left[1\;-1\;1\;\cdots \;-1\right]}_{N\times 1}$. For example, the shape vector of Sine-BOC(1,1) is $d={\left[1\;-1\right]}_{2}$, Tsc=Tc/2. Similarly, for Cosine-BOC(*m*,*n*), $N_{1}=4m/n$. The shape vector of Cosine-BOC(1,1) is $d={\left[1\;-1\;1\;-1\right]}_{4}$, Tsc=Tc/4.

The idea spreading code possesses the characteristic of $E[c_{i}c_{j}]=\delta_{ij}$. Hence, it can be applied into the baseband spread spectrum signal to achieve the correlation function:

(4) $$\begin{array}{ll} R(\tau ) & =E[s(t)s(t+\tau )]\\ & =\frac{1}{Tc}\sum_{i=-\infty }^{\infty }\sum_{j=-\infty }^{\infty }E[C_{i}C_{j}]\int_{0}^{Tc}P_{Tsc}(t-jTsc)P_{Tsc}(t+\tau -jTsc)dt\\ & =\frac{1}{Tc}\int_{0}^{Tc}sc(t)sc(t+\tau )dt\\ & =E[sc(t)sc(t+\tau )] \end{array}$$

In Equation (4), the autocorrelation of the spread spectrum signal that contains the idea spreading code is the same as the autocorrelation of the subcarrier modulated signal. Suppose that there are two subcarrier modulated signals that can respectively be denoted as:

(5) $$\left\{\begin{array}{c} s_{1}(t)=\sum_{j=0}^{N-1}d_{j}P_{Tsc}(t-jTsc)\\ s_{2}(t)=\sum_{j_{1}=0}^{N-1}d_{j_{1}}P_{Tsc}(t-j_{1}Tsc) \end{array}\right.$$

These have the same $f_{c}$ and N, but the shape vector $d_{j}$ and $d_{j_{1}}$ can be different. Therefore, the CCF obtained from the two subcarrier modulated signals can be written as:

(6) $$\begin{array}{ll} R_{CCF}(\tau ) & =E[s_{1}(t)s_{2}(t+\tau )]\\ & =\frac{1}{N}\sum_{j=0}^{N-1}\sum_{j_{1}=0}^{N-1}E[C_{j_{1}}C_{j}]\int_{0}^{Tc}P_{Tsc}(t-jTsc)P_{Tsc}(t+\tau -j_{1}Tsc)dt\\ & =\frac{1}{N}\sum_{j=0}^{N-1}\sum_{j_{1}=0}^{N-1}d_{j}d_{j_{1}}\Lambda_{Tsc}[\tau -(j-j_{1})Tsc] \end{array}$$

According to the characteristic of square waves ACF, we can deduce:

(7) $$\int_{-\infty }^{\infty }P_{Tsc}(t)P_{Tsc}(t+\tau )dt=\Lambda_{Tsc}(\tau ),$$

where $\Lambda_{Tsc}(\tau )$ means a triangle function, whose bottom width is 2Tsc, center is 0, and height is 1. Equation (6) can be applied into all BOC modulated signals to get correlation functions.

### 2.2. Ambiguous Problem

The ACF of the BOC modulated signal can be obtained by Equation (6), and the shape vectors $d_{j}$ and $d_{j_{1}}$ are identical. In [23], the ACF is given by:

(8) $$R_{\mathrm{BOC}(\mathrm{pn},n)}(\tau )=\left\{\begin{array}{l} {\left(-1\right)}^{k+1}(\frac{1}{p}(-k^{2}+2kp+k-p)-(4p-2k+1)\frac{\left|\tau \right|}{Tc}),\;\;\;\;\;\;\;\;|\tau |\leq kTc\\ 0,\;\;\;\;\;\;\;\;\;\;\;\;\;\;\;\;\;\;\;\;\;\;\;\;\;\;\;\;\;\;otherwise\; \end{array}\right.,$$

where $k=\left\lceil \frac{2p|\tau |}{Tc}\right\rceil$ with $i=\left\lceil x\right\rceil$ denoting the smallest integer so that i≥x.

**Figure 2:**
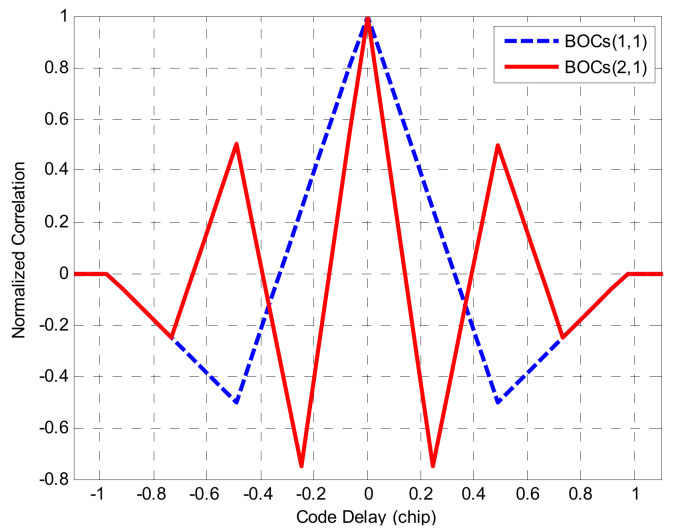
The auto-correlation functions (ACFs) of BOCs(1,1) and BOCs(2,1) signals.

In Figure 2, it is shown that it has side-peaks besides the main peak in the ACFs. In the signal synchronization process, the discriminator will appear as many zero-crossing points because of the multi-peak in the ACF, as a result, it may lock on one of the side peaks, which will cause a huge tracking error [24].

## 3. Proposed Unambiguous Synchronization Method

### 3.1. Method Design

According to the general formula of the BOC signal correlation function given by Equation (6), the BOC ACF is an image obtained by combining multiple shape code graphics. The shape code representation method of the BOCs(*m*,*n*) signal has been given in the previous section. Combining Equation (6), the correlation function of BOCs(*m*,*n*) expressed in matrix form can be obtained:

(9) $$R=\sum \left[\begin{array}{ccccc} d_{0}d_{0}\Lambda_{Tsc} & d_{1}d_{0}\Lambda (\tau -Tsc) & d_{2}d_{0}\Lambda (\tau -2Tsc) & \;\;\ldots \;\; & d_{N}d_{0}\Lambda (\tau -Tsc)\\ d_{0}d_{1}\Lambda (\tau +Tsc) & d_{1}d_{1}\Lambda_{Tsc} & d_{2}d_{1}\Lambda (\tau -Tsc) & \;\;\ldots \;\; & d_{N}d_{1}\Lambda (\tau -(N-1)Tsc)\\ d_{0}d_{2}\Lambda (\tau +2Tsc) & d_{1}d_{2}\Lambda (\tau +Tsc) & d_{2}d_{2}\Lambda_{Tx} & \;\;\ldots \;\; & d_{N}d_{2}\Lambda (\tau -(N-2)Tsc)\\ \;\;\vdots \;\; & \;\;\vdots \;\; & \;\;\vdots \;\; & \;\;\ddots \;\; & \;\;\vdots \;\;\\ d_{0}d_{N}\Lambda (\tau +NTsc) & d_{1}d_{N}\Lambda (\tau +(N-1)Tsc) & d_{2}d_{N}\Lambda (\tau +(N-2)Tsc) & \;\;\cdots \;\; & d_{N}d_{N}\Lambda_{Tx} \end{array}\right]$$

**Figure 3:**
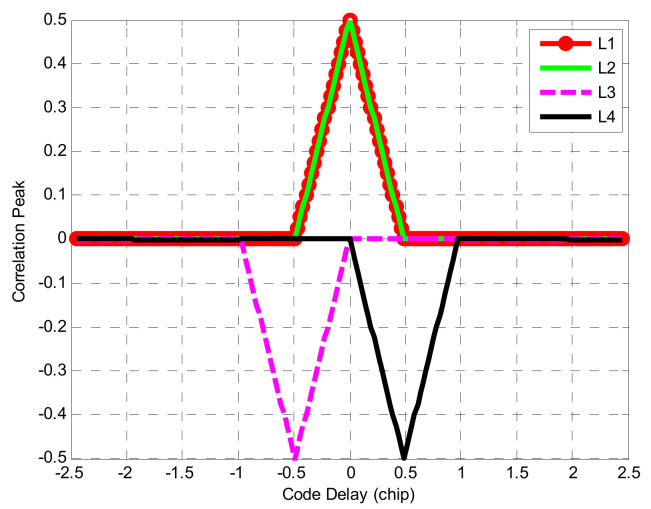
Composition of BOCs(1,1) signal autocorrelation function.

Equation (9) is an expansion equation of Equation (6). It can be seen from Equation (9) that the expression of the BOCs(*m*,*n*) ACF includes multiple trigonometric functions $\Lambda_{Tsc}$, where the peak value of any triangle Λ(τ−kTsc) is at the zero value of the adjacent triangle Λ(τ−(k−1)Tsc). The correlation function has the characteristic of piecewise linearity between any two peaks. Taking the BOCs(1,1) correlation function as an example, according to Equation (9), its shape code vectors are $d_{j_{1}}=[1,-1]$ and $d_{j_{2}}=[1,-1]$, and the corresponding correlation combinations are: $d_{j_{1}}0\cdot d_{j_{2}}0\cdot \Lambda_{Tsc}$, $d_{j_{1}}1\cdot d_{j_{2}}1\cdot \Lambda_{Tsc}$, $d_{j_{1}}0\cdot d_{j_{2}}1\cdot \Lambda (\tau +Tsc)$, and $d_{j_{1}}1\cdot d_{j_{2}}0\cdot \Lambda (\tau -Tsc)$, which are denoted as L1, L2, L3, and L4, respectively. The correlation combination of BOCs(1,1) is shown in Figure 3.

In Figure 3, L1 and L2 completely overlap. L3 and L4 are located below the X-axis respectively, and the four piecewise functions also have a common characteristic, which is symmetric with respect to the Y-axis. The two hypotenuses of L1 and L2 are parallel to the two hypotenuses of L3 and L4, respectively, so L1 + L3 and L2 + L4 are absolutely equal in amplitude. Mark L1 + L3 as C1, and L1 + L3 as C2. Combined with Figure 3, the specific shapes of C1 and C2 can be obtained.

Combining the above derivation process, it can be seen that in the matrix of Equation (9), the triangle formed by the shape code vector on the main diagonal is symmetric about the Y-axis, and Figure 3 shows that the graph obtained by a simple combination of symmetric graphs is also symmetric about the main diagonal. For the BOCs(*m*,*n*) signal, the shape code vector below the main diagonal is added as C1, and the addition above the main diagonal is recorded as C2. C1 and C2 are symmetric about the Y-axis, and these can be expressed as:

(10) $$R_{C1}(\tau )=R_{C2}(\tau )$$

**Figure 4:**
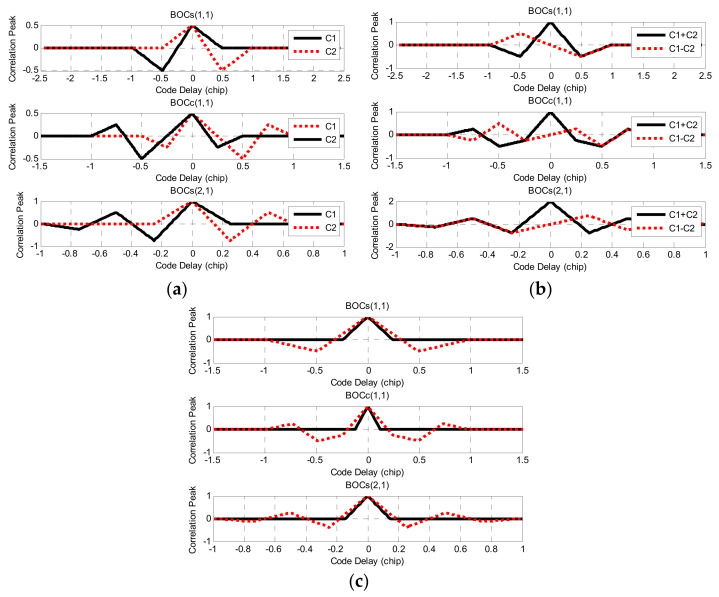
The reconstructed process of the BOCs(1,1), BOCc(1,1) and BOCs(2,1) signals: (**a**) The sub-correlation combination function; (**b**) The simple combination of sub-correlation function; (**c**) The reconstruction of correlation function.

The combined correlation functions C1 and C2 of BOCs(1,1), BOCc(1,1), and BOCs(2,1) are given, as shown in Figure 4a.

In Figure 4a, the combined correlation functions C1 and C2 of each BOC signal are symmetrical about the Y-axis, and the peak values of C1 and C2 are equal. This feature will play a crucial fulcrum part to remove BOC signal correlation ambiguity. The correlation functions C1 and C2 were mathematically added and subtracted to obtain the vector graphic combination shown in Figure 4b. C1 + C2 is the correlation function of the BOC signal itself. For BOC(1,1), there was only one narrow main peak at chip 0, and the amplitudes of the secondary peaks were equal at the ±0.5 chip. Similarly, for BOC(2,1), there was only one main peak at the 0 chip, and the amplitudes of the other sub-peaks were also equal. According to this characteristic, the designed reconstruction correlation function can be expressed as follows:

(11) $$R_{p}=\left|R_{C1}\right|+\left|R_{C2}\right|-\left|R_{C1}-R_{C2}\right|$$

According to Equation (11), the reconstructed BOC signal correlation function with edge peak elimination is shown in Figure 4c.

### 3.2. Waveform Design of Local Code

**Figure 5:**
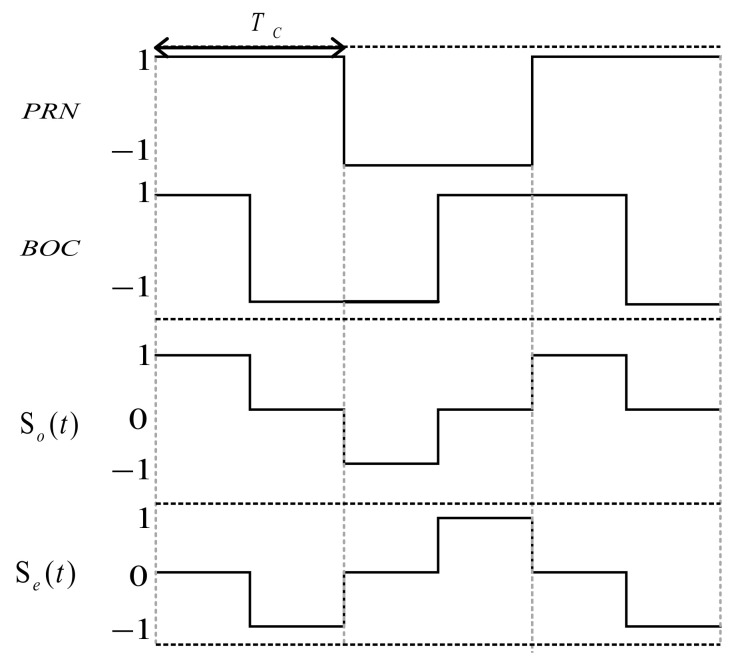
Local reference signal wave.

The study in [25] proposed an unambiguous method for Sine-BOC(2*n*,*n*) signals. Similarly, it showed a method for Sine-BOC signals in [26]. Some other papers have also undertaken some study on unambiguous synchronization, but it cannot be applied to Sine-BOC and Cosine-BOC signals at the same time. The proposed unambiguous method based on a reconstructed correlation function in this paper is feasible for sine-BOC and cosine-BOC signals and the principle is shown in Figure 5.

**Figure 6:**
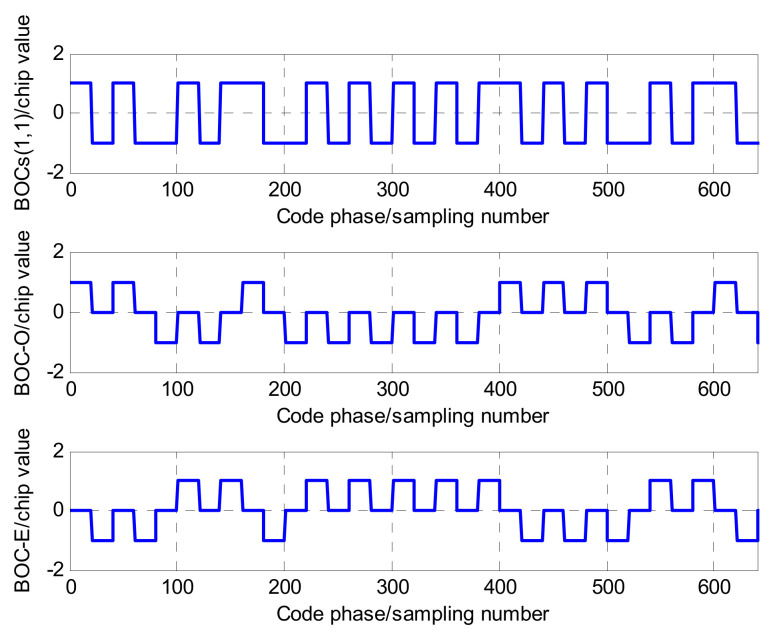
The separation of the BOCs(1,1) sequence.

**Figure 7:**
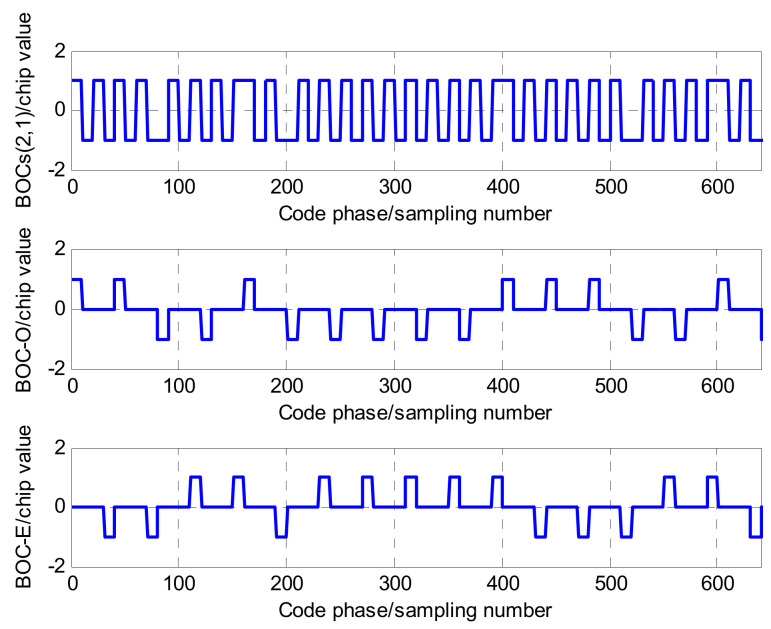
The separation of the BOCs(2,1) sequence.

In Figure 5, the local BOC signal is segmented into two units signals, referred to as two reference signals: one is the odd unit reference signal, denoted as $S_{o}(t)$ and the other is the even unit reference signal denoted as $S_{e}(t)$. The separation principle is as follows: for $S_{o}(t)$, the amplitude of the first sub-chip during the period of one spreading PRN code remains unchanged, and the remaining sub-chips become 0; similarly, for $S_{e}(t)$, the amplitude of the last sub-chip during the period of one spreading PRN code remains unchanged, and the remaining sub-chips become 0. Taking BOCs(1,1) and BOCs(2,1) as examples, Figure 6 and Figure 7 are the references signals based on the separation principle.

As seen in Figure 6 and Figure 7, two local reference signal sequences, $S_{o}(t)$ and $S_{e}(t)$, can be achieved by the separation of the local BOC signal. Their mathematical models can be respectively expressed as:

(12) $$\begin{array}{l} S_{o}(t)=\left\{\begin{array}{ll} \sum_{i=-\infty }^{+\infty }\sum_{j=0}^{N-1}C_{i}d_{j}P_{Tsc}(t-iTc-jTsc), & iTc\leq t\leq (iTc+Tsc)\\ \;0,\;\; & \;\;\left(iTc+Tsc)\leq t\leq (iTc+(N-1)Tsc\right) \end{array}\right.\\ S_{e}(t)=\left\{\begin{array}{ll} \;\;0,\;\; & iTc\leq t\leq (iTc+(N-2)TSC)\\ \sum_{i=-\infty }^{+\infty }\sum_{j=0}^{N-1}C_{i}d_{j}P_{Tsc}(t-iTc-jTsc), & \left(iTc+(N-2)Tsc)\leq t\leq (iTc+(N-1)Tsc\right) \end{array}\right. \end{array}$$

Combining the shape vector, they can be further derived as follows:

(13) $$\left\{\begin{array}{c} S_{o}(t)=\sum_{i=-\infty }^{+\infty }\sum_{j_{o}=0}^{N-1}C_{i}d_{jo}P_{Tsc}(t-iTc-j_{o}Tsc),\;\;\;\;\;d_{jo}={\left[0\;\;\;0\;\;\;0\;\;\;\cdots \;\;\;1\right]}_{N}\\ S_{e}(t)=\sum_{i=-\infty }^{+\infty }\sum_{j_{e}=0}^{N-1}C_{i}d_{je}P_{Tsc}(t-iTc-j_{e}Tsc),\;\;\;\;\;d_{je}={\left[1\;\;\;0\;\;\;0\;\;\;\cdots \;\;\;0\right]}_{N} \end{array}\right.$$

In Equation (13), it can be seen that two local reference signal sequences had the same mathematical expressions as the baseband spread spectrum signal. They had the same $f_{c}$, Tsc, and N, but the shape vector was different. For BOCs(*m*,*n*) signals, the shape vector of the odd reference signal sequence was $d_{jo}={\left[1\;\;\;\;0\;\;\;\;0\;\;\;\cdots \;\;\;0\right]}_{\frac{2m}{n}}$ and the even reference signal sequence was $d_{je}={\left[0\;\;\;\;0\;\;\;\;0\;\;\;\cdots \;\;\;1\right]}_{\frac{2m}{n}}$. For BOCc(*m*,*n*) signals, the shape vectors of the two local reference signal sequences were $d_{jo}={\left[1\;\;\;\;0\;\;\;\;0\;\;\;\cdots \;\;\;0\right]}_{\frac{4m}{n}}$ and $d_{je}={\left[0\;\;\;\;0\;\;\;\;0\;\;\;\cdots \;\;\;1\right]}_{\frac{4m}{n}}$, respectively. Furthermore, the local reference signal sequence based on idea spreading code PRN, is similar to the baseband spread spectrum signal. Therefore, it possesses the characteristic of idea spreading code in Equation (4).

The cross-correlation function (CCF) between the received BOC signals and two local reference signal sequences can be denoted as $R_{o}(\tau )$, $R_{e}(\tau )$, and expressed as:

(14) $$\left\{\begin{array}{c} R_{o}(\tau )=\int_{0}^{Tc}S(t)S_{o}(t-\tau )dt\\ R_{e}(\tau )=\int_{0}^{Tc}S(t)S_{e}(t-\tau )dt \end{array}\right.$$

Since S(t), $S_{o}(t)$, and $S_{e}(t)$ have the same $f_{c}$ and N, according to Equation (6), we can deduce that:

(15) $$\left\{\begin{array}{c} R_{o}(\tau )=\int_{0}^{Tc}S(t)S_{o}(t-\tau )dt=\frac{1}{N}\sum_{j=0}^{N-1}\sum_{j_{o}=0}^{N-1}d_{j}d_{j_{o}}\Lambda_{Tsc}[\tau -(j-j_{o})Tsc]\\ R_{e}(\tau )=\int_{0}^{Tc}S(t)S_{e}(t-\tau )dt=\frac{1}{N}\sum_{j=0}^{N-1}\sum_{je=0}^{N-1}d_{j}d_{j_{e}}\Lambda_{Tsc}[\tau -(j-j_{e})Tsc] \end{array}\right.$$

In Equation (15), the shape vectors of S(t), $S_{o}(t)$, and $S_{e}(t)$ are $d_{j}$, $d_{jo}$, and $d_{je}$, respectively. Their CCFs are composed of several triangle functions $\Lambda_{Tsc}(\tau -kTsc)$; for the odd unit, the correlation value of the position is:

(16) $$r_{o}=\left\{\begin{array}{c} \frac{1}{N}\sum_{j=0}^{N-k-1}d_{j+k}d_{jo}\;\;\;\;\;\;\;\;\;\;0\leq k\leq N-1\\ \frac{1}{N}\sum_{j=0}^{N+k-1}d_{j}d_{jo-k}\;\;\;\;\;\;\;\;\;\;1-N\leq k\leq 0 \end{array}\right.$$

The correlation function between two adjacent peaks of triangle functions $\Lambda_{Tsc}(\tau -kTsc)$ is linear, because the peak position of the triangle functions $\Lambda_{Tsc}(\tau -(k-1)Tsc)$ is the same as the position of the zero value point of the triangle function $\Lambda_{Tsc}(\tau -kTsc)$. Furthermore, the shape of CCF is determined by $r_{o}$, which is derived from shape vector $d_{j}$ and $d_{jo}$. Similarly, for the even unit, the cross-correlation is determined by $r_{e}$, derived from shape vector $d_{j}$ and $d_{je}$. For $r_{o}$ and $r_{e}$, they have the same shape vector. The shape vectors of two local reference signal sequences, $d_{jo}$ and $d_{je}$, are center symmetrical in the angle of the waveform. Therefore, the CCFs are also center symmetrical according to Equation (15).

(17) $$R_{o}(-\tau )=R_{e}(\tau )$$

**Figure 8:**
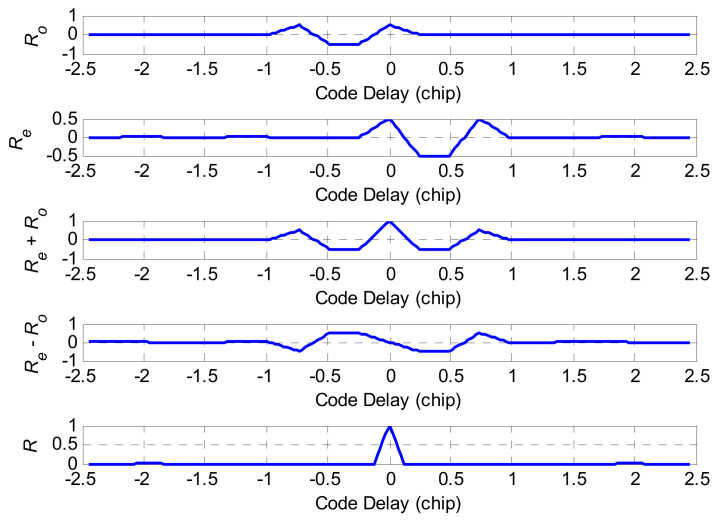
The reconstruction correlation function of the BOCc(1,1) signal.

**Figure 9:**
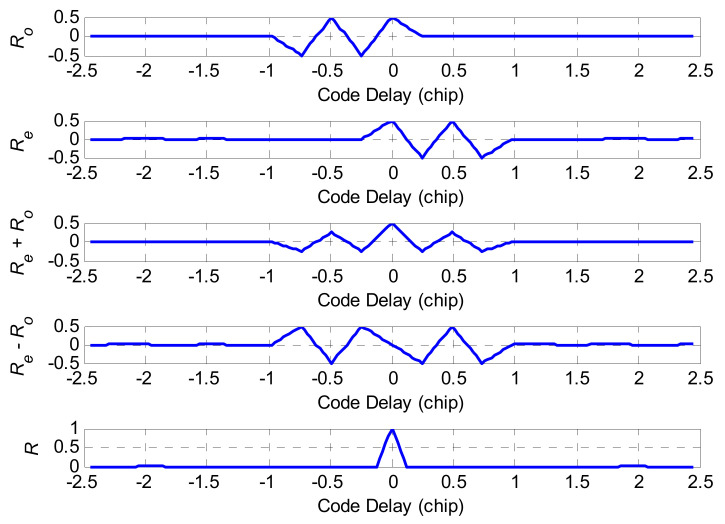
The reconstruction correlation function of the BOCs(2,1) signal.

The algorithm this paper proposed was implemented with the predetermined reconstruction rule:

(18) $$R=\kappa (|R_{e}+R_{o}|-|R_{e}-R_{o}|)$$

where κ is the reconstruction coefficient determined by κ=m/n. According to the reconstruction rule, Figure 8 and Figure 9 are the reconstruction correlation functions of the BOCc(1,1) and BOCs(2,1) signals, respectively. As shown in Figure 8 and Figure 9, there are still more than one peak in the $R_{e}(\tau )$ and $R_{o}(\tau )$, but they are center symmetrical. For $R_{e}+R_{o}$ and $R_{e}-R_{o}$, they have the same correlation value at the same code delay except the zero point, where the zero point of $R_{e}-R_{o}$ matches the main peak of $R_{e}+R_{o}$. Therefore, it obtains an ideal correlation function with the edge peaks wholly removed by subtracting the absolute value of $R_{e}+R_{o}$ from that of $R_{e}-R_{o}$, which can be expressed as:

(19) $$R=1-\frac{2|t|}{Tsc}$$

**Figure 10:**
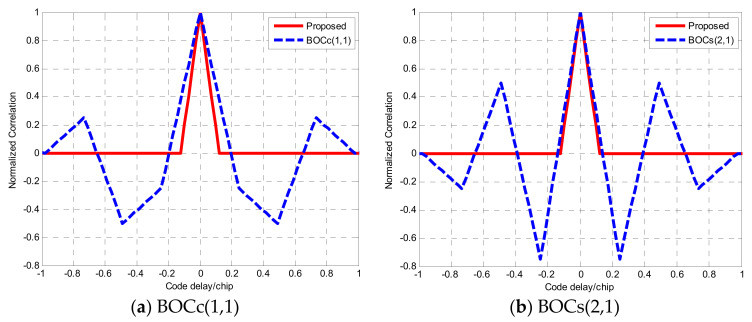
The reconstruction correlation function and comparison.

In Figure 10, the proposed method removes the side-peak and keeps the sharp main peak when compared with the ACF of the traditional BOCs(2,1) and BOCc(1,1) signal.

### 3.3. Tracking Loop Structure

In the tracking loop, the received intermediate frequency (IF) signal can be expressed as:

(20) $$S(t)=\sqrt{P_{s}}\times C(t-\tau )\times Sc(t-\tau )\times \cos[2\pi (f_{IF}+f_{D})]+n(t),$$

where $P_{s}$ is the power of input signal; C(t) is the PRN code; τ is the code delay of input signal; $f_{D}$ is the Doppler frequency of input signal; $\theta_{0}$ is the initial phase; $f_{IF}$ is the IF; Sc(t) is the subcarrier signal; and n(t) is the noise with the power spectral densities $N_{0}$.

After carrier cancellation and correlation, the in-phase and quadrature components of the received signal are given by Equation (21).

(21) $$\left[\begin{array}{c} S_{i}(t)\\ S_{q}(t) \end{array}\right]=\sqrt{P_{s}}C(t-\tau )Sc(t-\tau )\left[\begin{array}{c} \cos\varphi_{e}(t)\\ \sin\varphi_{e}(t) \end{array}\right]+\left[\begin{array}{c} n_{i}(t)\\ n_{q}(t) \end{array}\right],$$

where $\varphi_{e}(t)=2\pi f_{e}t+\theta_{e}$; $f_{e}$ is the frequency error; $\theta_{e}$ is the initial phase error; and $n_{i}(t)$ and $n_{q}(t)$ are independent zero-mean Gaussian noise components of n(t), with the same power spectral densities $N_{0}$.

**Figure 11:**
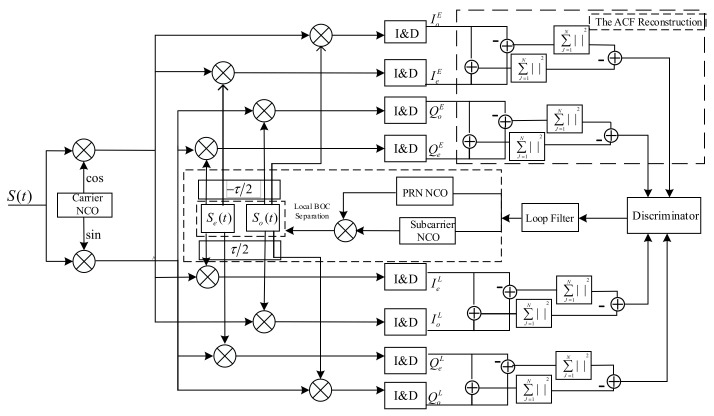
New delay tracking loop (DLL) architecture based on reconstruction correlation function.

According to the proposed method, we designed a new tracking loop. The new tracking loop structure is sketched in Figure 11. Compared with the traditional delay tracking loop (DLL), four additional correlators need to be added in the quadrature and in-phase branches. Through the locally generated auxiliary signal, an improved non-correlation discriminator that can realize no ambiguity tracking can be obtained. The received signal respectively correlates with two local reference signals $S_{e}(t)$ and $S_{o}(t)$, which are separated by the local BOC signal. Supposing that the carrier is wiped off, the CCFs of the received and the local reference signals are as follows:

(22) $$\left\{\begin{array}{c} I_{\mathrm{BOC}/j}^{E}=\sqrt{P_{s}}T_{P}R_{\mathrm{BOC}/j}(\varepsilon -\delta Tc)\cos(\theta_{e})+n_{I,j}^{E}\\ Q_{\mathrm{BOC}/j}^{E}=\sqrt{P_{s}}T_{P}R_{\mathrm{BOC}/j}(\varepsilon -\delta Tc)\sin(\theta_{e})+n_{Q,j}^{E}\\ I_{\mathrm{BOC}/j}^{L}=\sqrt{P_{s}}T_{P}R_{\mathrm{BOC}/j}(\varepsilon -\delta Tc)\cos(\theta_{e})+n_{I,j}^{L}\\ Q_{\mathrm{BOC}/j}^{L}=\sqrt{P_{s}}T_{P}R_{\mathrm{BOC}/j}(\varepsilon -\delta Tc)\sin(\theta_{e})+n_{Q,j}^{L} \end{array}\right.$$

*I* and *Q* are short for in-phase and quadrature components. The subscript j contains o and e, and they represent the odd reference signal and even reference signal. $R_{\mathrm{BOC}/j}$ is the CCF of the locally received signal and reference signal, ε is code delay; δTc represents the correlator spacing; $T_{P}$ is the integration time; and E and L represent the correlation location (early(E), late(L)). $n_{I,j}$ and $n_{Q,j}$ are the Gaussian noise.

**Figure 12:**
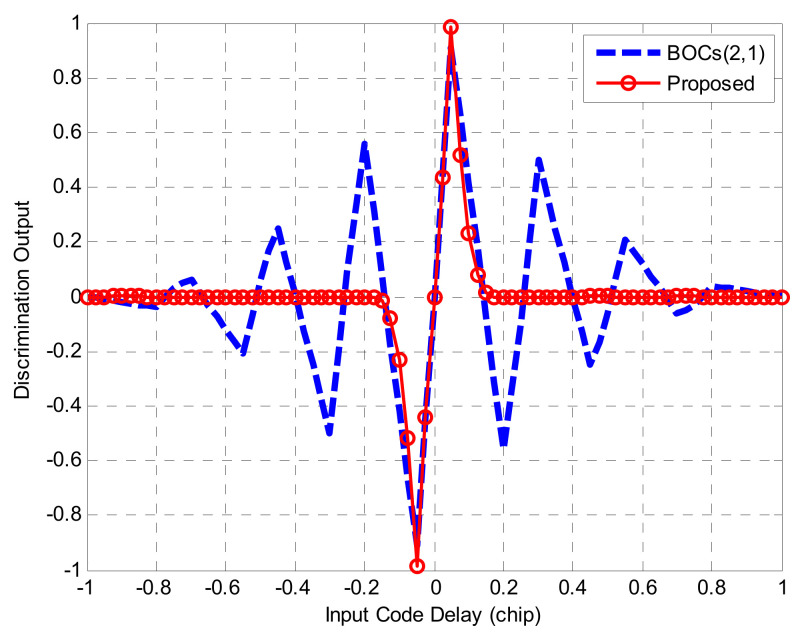
The reconstruction correlation function of the BOCs(2,1) signal.

Using the non-coherent early-late power (NELP) discriminator [27], depicted in Figure 12, the ultimate expression of the discriminator output is:

(23) $$\begin{array}{lll} D\left(\varepsilon \right) & = & {\left(|I_{BOC/e}^{E}+I_{\mathrm{BOC}/o}^{E}|-|I_{\mathrm{BOC}/e}^{E}-I_{\mathrm{BOC}/o}^{E}|\right)}^{2}+{\left(|Q_{\mathrm{BOC}/e}^{E}+Q_{\mathrm{BOC}/o}^{E}|-|Q_{\mathrm{BOC}/e}^{E}-Q_{\mathrm{BOC}/o}^{E}|\right)}^{2}-\\ & & {\left(|I_{\mathrm{BOC}/e}^{L}+I_{\mathrm{BOC}/o}^{L}|-|I_{\mathrm{BOC}/e}^{L}-I_{\mathrm{BOC}/o}^{L}|\right)}^{2}-{\left(Q_{\mathrm{BOC}/e}^{L}+Q_{\mathrm{BOC}/o}^{L}|-|Q_{\mathrm{BOC}/e}^{L}-Q_{\mathrm{BOC}/o}^{L}|\right)}^{2}\\ & = & P_{s}\times T_{P}^{2}\times (R^{2}(\varepsilon -\delta Tc)-R^{2}(\varepsilon -\delta Tc))+2\times \sqrt{P_{s}}\times T_{P}\times \{R(\varepsilon -\delta Tc)\times (n_{1,e}^{E}+n_{1,o}^{E})-\\ & & R(\varepsilon -\delta Tc)\times (n_{1,e}^{L}+n_{1,o}^{L})\}+{\left(n_{1,e}^{E}+n_{1,o}^{E}\right)}^{2}+{\left(n_{Q,e}^{E}+n_{Q,o}^{E}\right)}^{2}-{\left(n_{1,e}^{L}+n_{1,o}^{L}\right)}^{2}-{\left(n_{Q,e}^{L}+n_{Q,o}^{L}\right)}^{2} \end{array}$$

Based on Equation (23), ignoring the noise component in the discriminator output, Figure 10 shows this curve for the correlator spacing d=2δTc without the front-end filter. In order to clearly show the before and after improvement of the phase detector output, the discriminator curve using the NELP loop for the BOCs(2,1) autocorrelation function is shown in Figure 12. The proposed method removes the false lock points when compared with the BOCs(2,1) discriminator output curve.

### 3.4. Acquisition Structure Design

**Figure 13:**
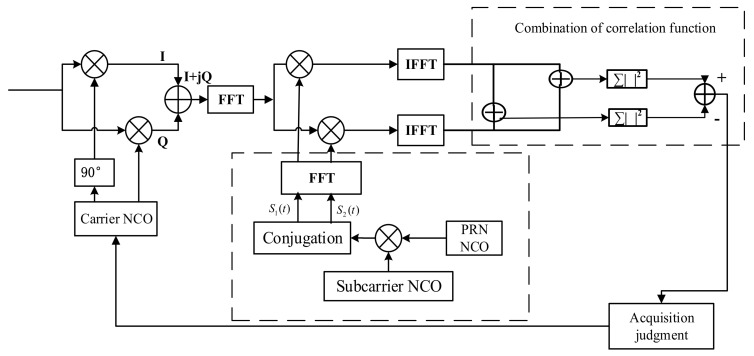
The acquisition structure schematic of the proposed method.

As can be seen from the above-mentioned Figure 13, in the acquisition principle block diagram of the proposed method, the GNSS receiver can use one correlation channel to accumulate the correlation integration results in the *i*th square wave range of each chip into a sub-function, therefore decreasing the computations and complex rate. If two code waveforms are generated, two related channels must be used. To eliminate the effect of the multi-peak, the quantity of calculation for generating 2-channel local codes will be appropriately increased.

### 3.5. Complexity Analysis

In the preset simulation environment, the received signal was sampled, and the data point N was 818,400. The range of the Doppler search was ±10 kHz, the search step length was set as f=500 Hz, and the frequency point of the Doppler search was $f_{bin}=21$.

The unit correlation algorithm and the proposed synchronization algorithm require five FFT operations, and the number of the complex multiplication operations and the real multiplication operations are both twice. ASPeCT and SCPC require eight FFT operations, four complex multiplications, and two real multiplications [15]. The BPSK-Like method requires six FFT operations, one complex multiplication, and four real multiplications [28]. According to the properties of FFT, one FFT operation is equivalent to $(N/2)\log_{2}N$ complex number multiplications and $N\log_{2}N$ complex number additions, one complex number multiplication equals four multiplications of two real numbers, and one complex number addition is two additions of real numbers (see Table 1 for the comparison of total calculations). The total calculated amount of the proposed algorithm was 41.46% of the ASPeCT algorithm and 57.32% of the BPSK-Like algorithm.

## 4. Performance Analysis

### 4.1. Acquisition Performance Simulation and Analysis

#### 4.1.1. De-Blurring Availability

To verify the generality and validity of the side-peak elimination algorithm, the simulation took BOC(1,1) and BOC(2,1) signals as examples using the ASPeCT [15] algorithm, SCPC [14] algorithm, and BPSK-like algorithm [10] for comparison.

**Figure 14:**
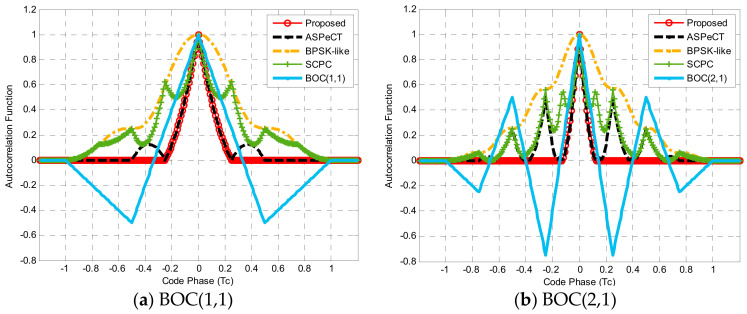
Comparison of the normalized autocorrelation functions.

In Figure 14, the simulation results showed that the BPSK-like and SCPC could eliminate the edge peaks of the ACF, but at the expense of the narrow main peak characteristic of the BOC signal, the obtained correlation function curve was also extremely unsmooth, which affected the signal tracking accuracy. The secondary peaks of the correlation function of ASPeCT were not completely eliminated. In the case of a weak signal, it is easy to cause a problem of false capture and false lock, which affects the positioning accuracy of the navigation receiver. In contrast, the proposed method not only completely eliminated the edge peaks, but also retained the advantage of narrow correlation of the BOC signal.

#### 4.1.2. Detection Probability

The synchronization process of the baseband signal of the GNSS receiver is divided into two steps: acquisition and tracking. The acquisition of the received signal is a procedure of constantly comparing the detection statistics output by the incoherent integrator with the detection threshold. The detection statistics value is the autocorrelation value or the square value of the amplitude output by the correlator, and the detection probability $P_{d}$ is a significant argument for the detection and acquisition property.

The uncorrelated detection statistics of the conventional acquisition scheme can be shown as:

(24) $$T=\sum_{j=1}^{M}\left(I_{j}^{2}+Q_{j}^{2}\right),$$

where

(25) $$\left\{\begin{array}{c} I_{j}=\sqrt{T_{s}C/N_{0}}\sin c\left(\pi \Delta f_{D}T_{s}\right)R(\Delta \tau )\cos\left(\Delta \varphi \right)+N_{i,j}\\ Q_{j}=\sqrt{T_{s}C/N_{0}}\sin c\left(\pi \Delta f_{D}T_{s}\right)R(\Delta \tau )\sin\left(\Delta \varphi \right)+N_{q,j} \end{array}\right.$$

where $T_{s}$ is the coherent integral time, and $C/N_{0}$ is the carrier-to-noise ratio. $\Delta f_{D}$, Δτ, and Δφ signify three types of errors, which are the Doppler frequency, code phase, and carrier phase, respectively. R(Δτ) denotes the CCF between the local code signal and the received signal. $N_{i,j}$ and $N_{q,j}$ are Gaussian white noise, where the mean value of both is 0 and the single sideband power spectral density of both is $N_{0}$.

Substituting Equation (25) into Equation (24), in this case, the detection statistic is:

(26) $$T=\sum_{j=1}^{M}\left[\begin{array}{c} {\left(\sqrt{T_{s}C/N_{0}}\sin c(\pi \Delta f_{D}T_{s})R(\Delta \tau )\cos\left(\Delta \varphi \right)+N_{i,j}\right)}^{2}+\\ {\left(\sqrt{T_{s}C/N_{0}}\sin c(\pi \Delta f_{D}T_{s})R(\Delta \tau )\sin\left(\Delta \varphi \right)+N_{q,j}\right)}^{2} \end{array}\right]$$

The detection statistic T observes the non-central $\chi^{2}$ chi-square distribution with the 2M degrees of freedom (DOF). The non-central arguments can be obtained:

(27) $$\zeta^{2}=MT_{s}C/N_{0}\sin c^{2}(\pi \Delta f_{D}T_{s})R^{2}(\Delta \tau )$$

Denote the probability density as $P_{T}(x)$, and the detection probability $P_{d}$ as:

(28) $$P_{d}=\int_{V}^{+\infty }P_{T}\left(x\right)dx$$

Among them, V is the detection threshold, which can be operated based on the provided false alarming probability $P_{fa}$. It can be seen from Equations (27) and (26) that only the code phase error tends to zero, and the statistical detection amount can reach the maximum.

The two locally generated waveforms $S_{e}(t)$ and $S_{o}(t)$ perform in-phase and quadrature operations with the receipt signal, respectively, which can be represented as:

(29) $$\left\{\begin{array}{c} I_{1,j}=\sqrt{T_{s}C/N_{0}}\sin c(\pi \Delta f_{D}T_{s})R_{1}(\Delta \tau )\cos(\Delta \varphi )+N_{1,i,j}\\ Q_{1,j}=\sqrt{T_{s}C/N_{0}}\sin c(\pi \Delta f_{D}T_{s})R_{1}(\Delta \tau )\sin(\Delta \varphi )+N_{1,q,j}\\ I_{2,j}=\sqrt{T_{s}C/N_{0}}\sin c(\pi \Delta f_{D}T_{s})R_{2}(\Delta \tau )\cos(\Delta \varphi )+N_{2,i,j}\\ Q_{2,j}=\sqrt{T_{s}C/N_{0}}\sin c(\pi \Delta f_{D}T_{s})R_{2}(\Delta \tau )\sin(\Delta \varphi )+N_{2,q,j} \end{array}\right.$$

where $I_{1,j}$ and $Q_{1,j}$ express the in-phase and quadrature correlated branch output of the $S_{e}(t)$ and the BOC signals, respectively. Assuming that the noise type added is Gaussian white noise, $I_{1,j}$($I_{2,j}$) and $Q_{1,j}$($Q_{2,j}$) are uncorrelated. Moreover, $I_{1,j}$($Q_{1,j}$) and $I_{2,j}$($Q_{2,j}$) are also uncorrelated, so this paper only had to prove that $N_{1,i,j}$ and $N_{2,i,j}$ were independent.

(30) $$\begin{array}{l} E[N_{1,i,j}N_{2,i,j}]=\frac{1}{T_{C}}\int_{0}^{T_{C}}n(t)s_{1}(t+\tau )dt\times \int_{0}^{T_{C}}n(v)s_{2}(v+\tau )dv\\ \;\;\;\;\;\;\;\;\;\;\;\;\;\;\;\;\;\;=\frac{N_{0}}{T^{2}}\int_{0}^{T_{p}}s_{1}(t)s_{2}(t+\tau )=\frac{N_{0}}{T^{2}}R_{12} \end{array}$$

In Equation (30), $R_{12}$ is the CCF of two local codes. Based on the designed two-channel local code shape code vectors, the CCF was 0, thus $N_{1,i,j}$ and $N_{2,i,j}$ were uncorrelated from the above derivation.

Combining the Equation (24) detection statistics equation and the reconstructed rules of correlation function, the detection statistics $T_{1}$ of the proposed method is as follows:

(31) $$T_{1}=\sum_{j=1}^{M}\left[\begin{array}{c} {\left(\left|I_{1,j}+I_{2,j}\right|-\left|I_{1,j}-I_{2,j}\right|\right)}^{2}+\\ {\left(\left|Q_{1,j}+Q_{2,j}\right|-\left|Q_{1,j}-Q_{2,j}\right|\right)}^{2} \end{array}\right]$$

As the $I_{1,j}$($Q_{1,j}$) and $I_{2,j}$($Q_{2,j}$) are uncorrelated, $T_{1}$ can be simplified to

(32) $$T_{1}=\sum_{j=1}^{M}\left[2(I_{1,j}^{2}+Q_{1,j}^{2})+2(I_{2,j}^{2}+Q_{2,j}^{2})\right]$$

Both $\sum_{j=1}^{M}\left(I_{1,j}^{2}+Q_{1,j}^{2}\right)$ and $\sum_{j=1}^{M}\left(I_{2,j}^{2}+Q_{2,j}^{2}\right)$ observed the non-central $\chi^{2}$ chi-square distribution with 2M DOF, and the non-central parameters can be derived as:

(33) $$\left\{\begin{array}{c} {\zeta_{e}}^{2}=2MT_{s}C/N_{0}\sin c^{2}(\pi \Delta f_{D}T_{s}){R_{1}}^{2}(\Delta \tau )\\ {\zeta_{o}}^{2}=2MT_{s}C/N_{0}\sin c^{2}(\pi \Delta f_{D}T_{s}){R_{2}}^{2}(\Delta \tau ) \end{array}\right.$$

Since the digital features of the detection statistics are usually inaccessible, the performance analysis was provided by simulating results. The comparison graph of the detection probability of BOC(1,1) and BOC(2,1) was simulated by the Monte Carlo method and a variety of acquisition methods were used for comparison. Among them, the false alarm probability $P_{fa}=10^{-6}$, the accumulation times *M* = 10, and the statistics of 20,000 times are as indicated below.

**Figure 15:**
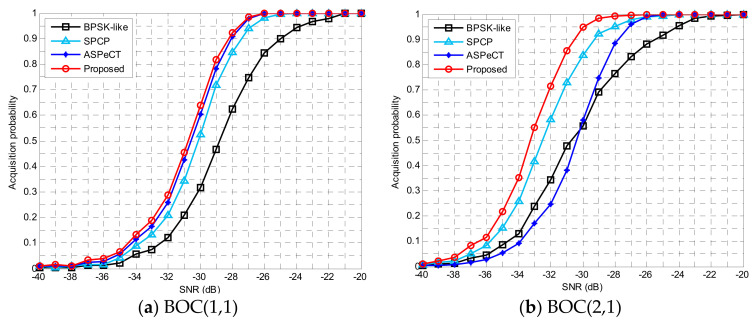
The acquisition probability.

In Figure 15, for the BOC(1,1) signal and BOC(2,1) signal, the detection probability of the proposed synchronization algorithm was significantly better than BPSK-like, SCPC, and ASPeCT. For BOC(1,1), the new unambiguous method could reach an acquisition probability that surpassed 90% at 35 dB. Compared with BPSK-like, SCPC, and ASPeCT, the acquisition sensitivity was improved by about 1.8, 0.7 and 0.2 dB, respectively. Similarly, for BOC(2,1), the detection sensitivity of the proposed was raised by about 1.7, 0.5 and 2.4 dB, respectively. As the modulation order increased, the superiority of the proposed scheme became more obvious.

#### 4.1.3. Peak-to-Average Ratio Analysis

**Figure 16:**
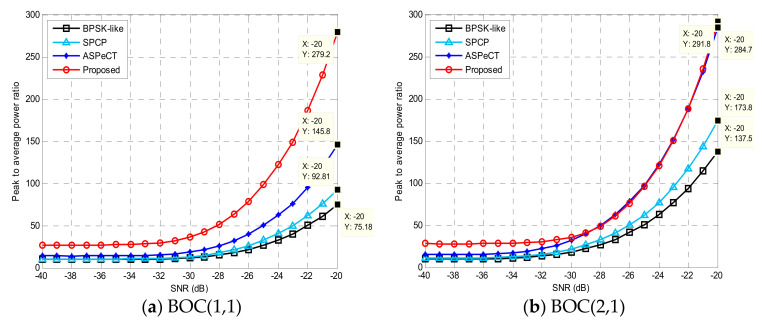
Peak-to-average power ratio.

Figure 16 revealed that the peak-to-average value and peak-to-peak ratio of signal acquisition also increased as the carrier-to-noise ratio (CNR) increased. Compared with several traditional acquisition algorithms, the improved algorithm had the highest peak-to-average value whether it was a second-order or fourth-order BOC signal. When the carrier-to-noise ratio was −20 dB, for the BOC(1,1) signal, the method in this paper was 204.02, 186.39, and 133.4 higher than BPSK-like, SCPC, and ASPeCT, respectively. For the BOC(2,1) signal, it was 154.3118 and 7.1 higher, respectively. This shows that the unambiguous algorithm in this paper had the highest peak-to-peak ratio and the best performance for unambiguous acquisition.

### 4.2. Tracking Performance Simulation and Analysis

#### 4.2.1. Anti-Noise Performance

In the tracking loop, the tracking performance can be described by the code error variance in the presence of thermal noise. The code error variance caused by traditional DLL was calculated in [28,29] for BPSK. The code error variance for the traditional BPSK signal is given by:

(34) $$d=2\delta Tc\sigma_{\varepsilon }^{2}=E[\frac{\varepsilon^{2}}{T_{C}^{2}}]=\frac{2B_{L}T_{P}R_{N}(0)}{\left(KTc\right)}$$

where $B_{L}$ is the code tracking loop single-sided bandwidth; $T_{P}$ is the coherent integral time; K is the phase discriminator gain; Tc is the code period; and $R_{N}(0)$ is the noise power. The same method can be applied for BOC modulation. According to Equation (19) and Equation (23), the difference coefficient at the zero point and the gain of the phase discrimination output curve can be deduced as:

(35) $$\begin{array}{ll} K & =[P_{s}T_{P}^{2}(R^{2}(\varepsilon -\delta Tc)-R^{2}(\varepsilon +\delta Tc)){]}_{\varepsilon =0}^{\prime }\\ & =-\frac{4P_{s}T_{P}^{2}}{Tc}(1-\delta \frac{4m}{n})(\frac{4m}{n}) \end{array}$$

Similarly, in Equation (23), since there is inevitably thermal noise in the discriminator, the error caused by it can be deduced as:

(36) $$\begin{array}{ll} n(t) & =2\sqrt{P_{s}}T_{P}(R(\varepsilon -\delta Tc)(n_{I,e}^{E}+n_{I,o}^{E})-R(\varepsilon +\delta Tc)(n_{I,e}^{L}+n_{I,o}^{L}))+\\ & =(n_{I,e}^{E}+n_{I,o}^{E}{)}^{2}+(n_{Q,e}^{E}+n_{Q,o}^{E}{)}^{2}-((n_{I,e}^{L}+n_{I,o}^{L}{)}^{2}+(n_{Q,e}^{L}+n_{Q,o}^{L}{)}^{2}) \end{array}$$

Thus, the variance of thermal noise can be calculated by the following formula:

(37) $$\begin{array}{lll} R_{N}(t) & = & E[n(t)n(t+\tau )]\\ & = & 4P_{s}T_{P}^{3}N_{0}R^{2}(\varepsilon +\delta Tc)\{R(\varepsilon )-R(\varepsilon +2\delta Tc)\}+\\ & & N_{0}T_{P}^{2}\{R^{2}(\varepsilon )-R^{2}(\varepsilon +2\delta Tc)\} \end{array}$$

Substituting Equation (35) and Equation (37) into Equation (34), and assuming the E–L spacing equals d=2δ, the code variance of this paper can be derived as:

(38) $$\sigma_{\varepsilon }^{2}=\frac{B_{L}d}{2\left(\frac{4m}{n}\right)C/N_{0}}\left(1+\frac{1}{\left(1-\frac{d}{2}\times \frac{4m}{n}\right)(C/N_{0})T_{P}}\right)$$

Thermal noise is one of the major reasons for the decrease in code tracking accuracy. Code tracking error standard deviation is a vital index to evaluate the pros and cons of the noise resistance of tracking algorithms [30]. According to Equation (38), several figures were plotted to simulate the curve of the code tracking error variance with the variation of the carrier-to-noise ratio. Assuming that the code tracking loop uses a NELP discriminator and the correlator E–L spacing is d=0.1 chip, the single-sided loop bandwidth $B_{L}=2\;\mathrm{Hz}$ and $T_{coh}=1\;\mathrm{ms}$.

**Figure 17:**
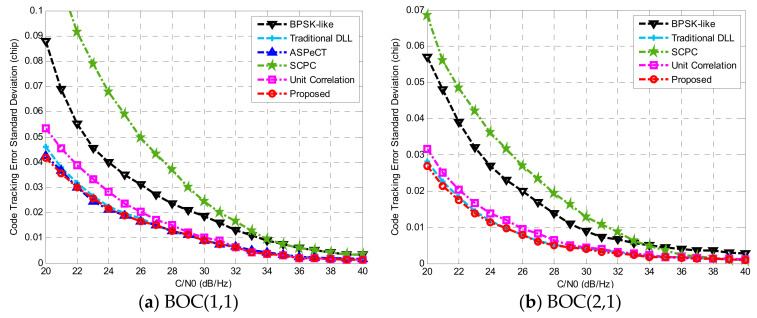
Code tracking error standard deviation.

Figure 17a shows the code tracking error standard deviation [23] simulated by several tracking methods to track the BOC(1,1) signal under different carrier-to-noise ratio (CNR). Among them, the code tracking error of BPSK-like and SCPC was relatively large. The code tracking error of ASPeCT, unit correlation, and the method in this paper were closer to the result obtained by the traditional DLL phase detector. Additionally, there was a certain improvement in the case of low C/N ratio.

Figure 17b gives the tracking result for BOC(2,1) when $B_{L}=2\;\mathrm{Hz}$, $T_{coh}=1\;\mathrm{ms}$, and the correlator interval is 0.05 Tc. As ASPeCT only applies to BOC(*n*,*n*), we will not analyze it here. The code tracking error obtained by BPSK-like and SCPC methods was still relatively large. The proposed method was close to the result obtained by the traditional DLL phase detector. For BOC(1,1) and BOC(2,1), compared with the SCPC, code tracking error standard deviation of the proposed method reduced 0.088 *Tc* and 0.042 *Tc*, respectively, indicating that the anti-noise performance of this method has been greatly improved. In addition, this method can almost apply to BOC modulated signals of any order.

#### 4.2.2. Phase Discrimination Curve

Equation (23) gives the NELP (non-coherent early-late power) phase detection formula of the proposed method, and the following simulation rests on an assumption that the front-end bandwidth is limitless.

**Figure 18:**
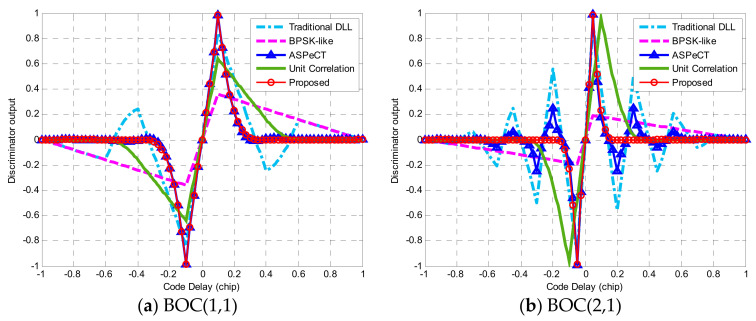
The phase discrimination curve.

Figure 18a shows the phase discrimination output results of BOC(1,1) processed by traditional DLL, BPSK-like, ASPeCT, unit correlation [31], and the proposed method in this paper when the correlator spacing was 0.1 *Tc*. Figure 18b shows the phase discrimination output of each method to BOC(2,1) when the correlator interval is 0.05 *Tc*.

The results show that for BOC(1,1), the phase detection curve of conventional DLL had two mis-lock points, and all four algorithms were able to take the false lock points out. In addition to the unit correlation algorithm, the stability regions of the other four phase detection curves were all (−0.1 *Tc*, +0.1 *Tc*), and the slope gain of the discrimination curves relative to the linear region of BPSK-like was 5.2 dB. For BOC(2,1), using the traditional DLL approach, the false lock points of the phase discriminator output curve turned into six points. Except for the unit correlation algorithm, the stable region of each algorithm was (−0.05 *Tc* + 0.05 *Tc*). ASPeCT has four false locking points, which indicates that it does not have the ability to erase synchronization blurring availability. In contrast, the proposed method wholly eliminates the false lock points, and the relative BPSK-like slope gain was 7.2 dB. Therefore, the proposed method can not only remove all the false lock points of the phase detection curve, but also maintain the larger slope of BOC(*kn*,*n*) obtained in the traditional DLL.

#### 4.2.3. Impact of Multipath

The studies in [32,33,34] discussed some techniques to mitigate the code/carrier multipath. To investigate the influence of multipath in the process of tracking, we took into account a ordinary model of multipath as a one-path specular refection, had some amplitude relative to the direct path, and arrived at the same phase and delay. As we all know, in the presence of the multipath, the output curve of discriminator is changed, especially the zero-crossing point, and thus causes a tracking error. The multipath error envelope (MEE) is a representative index to measure the multipath mitigation capability [35].

**Figure 19:**
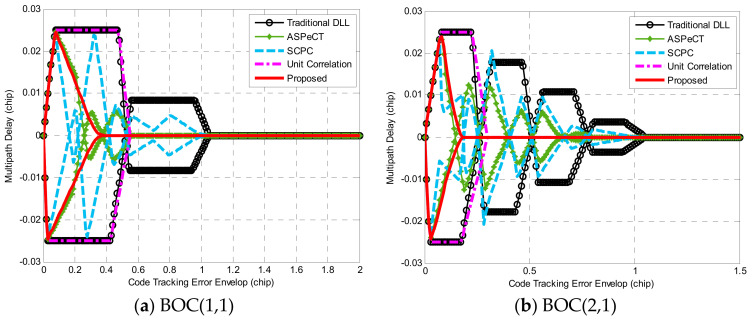
Comparison of the multipath error envelope (MEE) curves.

Figure 19a,b display the MEE curves comparison of BOC(1,1) and BOC(2,1), respectively, obtained through different synchronization algorithms when the correlator interval was 0.1 Tc. The envelope extremum value refers to the maximum absolute value of MEE, the envelope range is the sum of the MEE non-zero value spacing along the x-coordinate, and the envelope area is the size of the graph enclosed by MEE, which were applied to appraise the pros and cons of multipath interference [36]. The smaller the three indicators, the better the ability against multi-path interference.

It can be seen from Figure 19 that the multipath mitigation performance of traditional DLL was the worst. The anti-multipath performance of ASPeCT and SCPC was equivalent to the proposed method under the condition of short multipath delay range. However, ASPeCT did not absolutely erase the secondary peaks, resulting in errors in the mid-to-high multipath delay range. Although SCPC completely removed the secondary peak, it sacrifices the narrow correlation characteristic of the BOC signal. Therefore, in the medium and long delay range, the multipath mitigation performance of the two was poor. The multipath envelope area and interval length of the unit correlation method were both larger than the proposed methods. For BOC(1,1) and BOC(2,1), the three anti-multipath performance evaluation indexes obtained by the proposed method all had the smallest value among the above methods, so the proposed method possesses optimal ability against multipath interference.

## 5. Conclusions

Synchronization ambiguity of BOC signals is a significant problem for modern high accuracy GNSS applications. Aiming at the ambiguity of acquisition and tracking, an unambiguous reconstructed single sharp peak correlation function was obtained in this paper. Compared with several traditional methods, the proposed synchronization technique had more robust tracking, could provide excellent acquisition performance, and also simplify the synchronization architecture. Theoretical analysis and simulation proved that the proposed method removed the interference of the side-peak completely. The proposed method enables BOC and its derivative signals to be better applied to the new generation of navigation systems. In terms of acquisition performance, the detection probability and peak-to-average ratio of the proposed method were superior to the traditional algorithms. In terms of tracking capacity, the code tracking error of the proposed technique was relatively minimum, and the extremum, the interval length, and the envelope area of MEE were all smaller than the other algorithms. In addition, the technique had good anti-multipath capability, which can decrease the energy loss and signal distortion caused by the space-time filtering process to the BOC signal, and achieve the goal of reducing the positioning deviation of the navigation system as much as possible.

## Figures and Tables

**Table 1:** The calculation burden between different algorithms.

Method Name	Multiplication/Times	Addition/Times
Proposed method	$10N\log_{2}N+10$	$7N\log_{2}N+7$
Unit correlation	$10N\log_{2}N+10$	$7N\log_{2}N+7$
ASPeCT/SCPC	$16N\log_{2}N+18$	$24N\log_{2}N+8$
BPSK-like	$12N\log_{2}N+6$	$18N\log_{2}N+2$

## Data Availability

Not applicable.
